# Multi-target regulatory mechanisms and clinical assessment of natural products for insomnia: a review

**DOI:** 10.3389/fphar.2026.1681709

**Published:** 2026-01-14

**Authors:** Dongchuan Ma, Hao Yang, Maoyuan Zhao, Zhaoliang Luo, Renyi Teng, Xiaofei Huang, Tingting Liu, Shangbin Lv, Mingyu Wang

**Affiliations:** 1 Chongqing Traditional Chinese Medicine Hospital, Chongqing, China; 2 Beijing University of Chinese Medicine, Beijing, China; 3 Andingmen Traditional Chinese Medicine Hospital, Beijing, China; 4 Jiangjin Hospital of Traditional Chinese Medicine, Chongqing, China; 5 People’s Hospital of Shapingba District Chongqing, Chongqing, China; 6 Chongqing University of Chinese Medicine, Chongqing, China; 7 Chongqing Academy of Chinese Materia Medica, Chongqing, China

**Keywords:** clinical research, GABA, Glu, insomnia, natural products, traditional Chinese medicine formulations

## Abstract

**Background:**

Insomnia, a prevalent sleep disorder, adversely impacts patients’ quality of life and imposes significant burdens on both physical and mental health. While conventional insomnia therapies remain widely utilized, they exhibit persistent safety limitations, including risks of dependence and cognitive impairment. Natural products have garnered increasing scientific interest owing to their favorable safety profiles and demonstrated therapeutic efficacy. This comprehensive review critically examines contemporary advances in understanding the mechanistic actions of natural products against insomnia and their supporting clinical evidence, with the objective of synthesizing their pharmacological mechanisms, clinical effectiveness, and safety evaluations.

**Methods:**

This systematic review retrieved relevant literature through comprehensive searches of four core biomedical databases (ScienceDirect, PubMed, Ovid MEDLINE, and Web of Science), encompassing peer-reviewed articles published between January 2020 and August 2025. The retrieval strategy focuses on identifying original research papers concerning the treatment of insomnia using purified natural products and traditional Chinese medicine compound formulations.

**Results:**

Natural products demonstrate significant therapeutic efficacy against insomnia through multi-target mechanisms, including: modulation of GABAergic neurotransmission, regulation of orexinergic signaling pathways, attenuation of inflammatory responses and oxidative stress, restructuring gut microbiota composition, and normalization of core circadian regulators (notably CLOCK/BMAL1 complexes). Critically, clinical evidence confirms these natural products treatment outcomes with favorable safety profiles in insomnia management.

**Conclusion:**

This review critically evaluates contemporary advances in botanical therapeutics for insomnia management, synthesizing evidence to inform evidence-based clinical translation and facilitate the development of novel pharmacotherapeutic strategies.

## Introduction

Insomnia is a prevalent sleep disorder characterized by difficulty initiating sleep, sleep maintenance difficulties, and early morning awakening, often accompanied by impaired daytime functioning. Research indicates that insomnia not only affects individuals’ daily lives but may also lead to serious health issues such as cardiovascular diseases, metabolic disorders, and psychiatric conditions (Sutton, 2021; Manolis et al., 2021). Particularly among young adults, the association between insomnia and mental health problems has garnered increasing attention, with studies revealing elevated cardiovascular event risks in young patients that are closely linked to psychological disturbances (Park et al., 2023). Consequently, developing effective treatments for insomnia is critically important.

Currently, pharmacotherapy remains the primary approach for insomnia management. However, traditional sedative-hypnotic medications such as benzodiazepines, while effective, are often associated with dependency and adverse effects (Albrecht et al., 2014; Gaudreault et al., 1991; Murphy et al., 2016). Consequently, increasing research has focused on the potential of natural products, whose multi-target, low-toxicity, and high-safety profiles make them emerging candidates for insomnia treatment (Hosseini et al., 2024; Zhao F. Y. et al., 2023; Frost et al., 2024). For instance, studies indicate that certain botanical drug like valerian and chamomile are widely used in adjunctive insomnia therapy, with their efficacy gradually receiving empirical support (Zhao F. Y. et al., 2023).

In addition, the biological mechanisms of insomnia have also attracted widespread attention from researchers. The pathogenesis of insomnia involves a complex interplay among systemic inflammation, intestinal microbiota dysbiosis, and neuronal impairment. Chronically elevated pro-inflammatory cytokines (e.g., IL-1β, TNF-α) disrupt blood-brain barrier integrity, facilitating neuroinflammation that compromises sleep-regulating nuclei in the hypothalamus (Ballesio, 2023; Veler, 2023). Concurrently, gut dysbiosis-driven reductions in microbial metabolites (e.g., short-chain fatty acids) impair GABAergic neurotransmission while increasing circulating lipopolysaccharides that activate microglial-mediated neurotoxicity (Feng et al., 2023). This tripartite cascade ultimately induces structural and functional alterations in prefrontal-limbic circuits–particularly hippocampal neurogenesis suppression and amygdalar hyperactivation–which perpetuate hyperarousal states central to insomnia pathophysiology. For example, certain natural products can regulate neurotransmitter levels, thereby improving sleep quality (Cheng et al., 2024). These findings provide an emerging potential for the application of natural products in the treatment of insomnia.

In summary, the prevalence of insomnia and its health implications prompt a reevaluation of the limitations associated with conventional pharmacotherapy. As an alternative therapeutic approach, natural products exhibit considerable potential in insomnia management owing to their multitarget mechanisms of action and favourable safety profile (Frost et al., 2024). This study reviews 5 years of fundamental research and clinical trials on natural products for treating insomnia, confirming their efficacy in managing the condition and elucidating their primary mechanisms of action. It aims to provide patients with safer therapeutic options. This review pioneers the demonstration of how natural products recalibrate neurotransmitters, inflammation, gut microbiota, and circadian rhythm disorders through polypharmacological targets, delivering therapeutic effects unattainable by conventional receptor-overactivating hypnotics.

## Biological mechanisms of insomnia

### Disorder of the neurotransmitter system causes insomnia

Disruption of the neurotransmitter system constitutes the core pathophysiological mechanism of insomnia, primarily manifested as weakened GABAergic inhibition and enhanced glutamatergic excitation within sleep-wake regulatory nuclei (Bonanno et al., 2006). This neurochemical imbalance destabilizes thalamocortical circuits and the homeostasis of the ventrolateral preoptic nucleus (VLPO), resulting in diminished sleep drive and heightened hyperarousal (Arrigoni and Fuller, 2022). Notably, chronic insomnia is characterized by reduced GABA A receptor sensitivity in the anterior hypothalamus alongside elevated orexinergic activity in the lateral hypothalamus. These alterations collectively increase cortical excitability and interfere with sleep spindle generation—an electrophysiological hallmark of sleep maintenance. The interaction between GABAergic and serotonergic (5-HT) systems critically modulates insomnia pathogenesis through dysregulation of sleep-wake transition mechanisms (Li, 2020). Overexpression of 5-HT receptors in the thalamic reticular nucleus enhances cortical excitability *via* glutamatergic disinhibition (Arias et al., 2025). Weakened GABA B receptor-mediated inhibition of 5-HT neurons elevates peripheral 5-HT levels and impairs 5-HT auto receptor feedback (Yu et al., 2022). The “paradoxical” co-release of glutamate during non-rapid eye movement (NREM) sleep disrupts spindle formation and intensifies hyperarousal, thereby establishing a self-sustaining pathological circuit in chronic insomnia (Machida et al., 2021; Cheng et al., 2020). Additionally, orexin is a neuropeptide specifically synthesized by neurons in the lateral hypothalamic area (LHA) and serves as a critical regulator of sleep. Orexin influences the sleep-wake cycle and stress response through interactions with orexin receptors. The orexin/orexin R signaling cascade demonstrates complex neuroprotective and anti-inflammatory effects by inhibiting nuclear factor-κB (NF-κB) and PLC/Ca^2+^ pathway activation. Overexpression of orexin in individuals leads to sleep deprivation and circadian rhythm disruption (Palagini et al., 2022; Panda et al., 2025).

### Oxidative stress and inflammation promote insomnia

Oxidative stress and neuroinflammation synergistically drive the pathological progression of insomnia (Palagini et al., 2022). Under pathological conditions, reactive oxygen species (ROS) accumulate abnormally in the brain’s sleep-regulating regions, thereby impairing mitochondrial function in GABAergic neurons of the ventrolateral preoptic area (VLPO) of the hypothalamus. When mitochondrial function is compromised, ATP synthesis is reduced, subsequently inhibiting neuronal activity and diminishing sleep (Bin Heyat et al., 2022). Concurrently, the TLR4/NF-κB pathway in microglia undergoes sustained activation by damage-associated molecular patterns (DAMPs), thereby promoting elevated expression of IL-1β and tumour necrosis factor-α TNF-α (Zhang M. et al., 2025; Huang et al., 2019). These pro-inflammatory factors not only inhibit GABA but also cause abnormal melatonin secretion (Zisapel, 2012; Wang et al., 2015). Notably, inflammation-induced NADPH oxidase activation further amplifies oxidative stress, while ROS-mediated assembly of the NLRP3 inflammasome exacerbates IL-1β release, ultimately causing structural damage to the sleep homeostasis regulatory network. This manifests as significantly reduced δ-wave power during non-rapid eye movement (NREM) sleep and increased frequency of microarousals (Wang F. et al., 2022; Vlad et al., 2025; Zielinski et al., 2017).

### Circadian gene abnormalities affects sleep

The core circadian clock gene network regulates sleep through transcriptional control of sleep-wake effector systems (He et al., 2023). The CLOCK-BMAL1 transcription complex serves as a central regulator of sleep-wake rhythms (Iweka et al., 2023; Mendlewicz, 2009). This complex maintains circadian homeostasis by activating clock genes such as PER and CRY, while phosphorylation of BMAL1 enhances its transcriptional activity. Haploinsufficiency of the BMAL1 gene reduces PER2 oscillation amplitude in the suprachiasmatic nucleus (SCN), delays the peak time of melatonin secretion, and prolongs sleep onset latency (Crnko et al., 2023; Torres-Farfan et al., 2011). Reduced p-BMAL1 expression weakens the suppression of NF-κB, driving neuroinflammation and impairing GABAergic neuronal function. Disrupted rhythmic output signals cause melatonin secretion disorders and reduced slow-wave sleep during non-rapid eye movement (NREM) sleep, ultimately manifesting as sleep fragmentation and increased microarousals (Jahanban-Esfahlan et al., 2018; Li J. et al., 2024; Li Y. et al., 2024). This cascade constitutes the fundamental molecular basis of insomnia pathogenesis.

### Gut microbiota regulation of insomnia

The gut microbiota plays a significant role in the pathogenesis of insomnia by directly and indirectly regulating sleep behavior through the microbiota-gut-brain axis (Wang Z. et al., 2022). This signalling pathway transmits microbial signals to the brain, subsequently relaying signals generated by the brain to intestinal epithelial cells (Du et al., 2024). Signals propagate through the enteric nervous system - comprising 100–500 million neurons in the submucosal and myenteric plexuses - to reach gut microbes. Short-chain fatty acids (SCFAs) produced by gut bacteria bind free fatty acid receptors on intestinal epithelial cells, subsequently interacting with neurons or entering systemic circulation (Dicks, 2022). Indoleamine 2,3-dioxygenase derived from *Bacteroides* diverts tryptophan toward indoleamine synthesis rather than serotonin production, reducing serotonin bioavailability (Deng et al., 2021). Disrupted SCN circadian synchronization occurs due to impaired histone deacetylase inhibition following depletion of butyrate-producing Roseburia, hindering PER2 chromatin remodeling and causing abnormal melatonin secretion (Song et al., 2021). Lipopolysaccharide activates TLR4 on hypothalamic astrocytes, triggering TNF-α-mediated inflammation, which leads to a significant reduction in GABA synthesis (Zhang et al., 2022; Zhou and Hong, 2021) to induce insomnia.

## Natural products with hypnotic effects

In this review, research papers on the effects of natural products on sleep disorders were collected from various databases (including Scopus, PubMed, Medline, and Web of Science) up to August 2025, covering nearly 5 years, and were included in the current review article (Tables 1 and 2). The search terms for this study comprised “Insomnia OR Sleep disorder” AND “natural product OR natural OR Natural preparation OR natural compound OR phytochemical.” Literature retrieval was conducted by pairing these search terms in various combinations. Research papers investigating the use of natural products for treating insomnia were screened. Ultimately, 34 target papers were selected.

**TABLE 1:** Pharmacological research on natural products for treating insomnia.

Natural products for treating insomnia				
Extract/Formula	Study design	Dosage	Mechanism	Result
Jujuboside A	*In vivo*, C57 BL/6J mice	9, 18 mg/kg/d, 7 days, i.g.	↑GABA, ↓GLU, ↑GABA A, ↑GABA B, ↓mPTP, ↓Cyt c, ↑ATP	↑Sleep duration ↓Sleep latency
*Humulus lupulus* L. extract	*In vivo*, ICR mice	100, 200 mg/kg, 1 h, i.g.	↑GABA A, ↑GABA, ↑5-HT	↑Sleep duration ↑Sleep quality
Rhizome of *Nelumbo nucifera* Gaertn. extract	*In vivo*, ICR mice	80, 100, 120, 150 mg/kg, 1 h, i.g.	↑GABA A, ↑GABA,	↑Sleep duration ↑Sleep quality
Lavender	*In vivo*, C57 BL/6J mice	25.0%, 2 h, i.g.	↑GABA	↑Sleep duration ↓Sleep latency
*Mulberry leaf* (Folium Mori) extract	*In vivo*, KM mice	50, 100, 200 mg/kg/d, 7 days, i.g.	↑GABA, ↑5-HT	↑Sleep duration ↓Sleep latency
*Panax ginseng* extract	*In vivo*, ICR mice	7.5, 15, 30 mg/kg/d, 28 days, i.g.	↓Orexin, ↓PI3K, ↓Akt, ↓mTOR, ↑LC3A/B	↑Sleep duration Improve cognitive function Repair hippocampal neurons
Periostracum cicadae	*In vivo*, SD rat	4.568, 18.272 mg/mL/d, 7 days, i.p.	↑5-HT1A, ↑BDNF, ↑DARPP-32	↓Total distance ↓Average speed ↑Stationary time
*Rosmarinus officinalis* L. extract	*In vivo*, SD rat	10, 12.5, 15 g/kg/d, 6 days, i.g.	↑GABA, ↑5-HT, ↑DA, ↑5-HT1AR, ↑PKA, ↑GABAA, ↑cAMP, ↑ADCY5	Improvement of circadian rhythm
Sedum kamtschaticum extract	*In vivo*, SD rat	30 mg/kg, 1 h, i.g.	↑A_2A_ receptor, ↑GABA	↑Sleep duration ↓Sleep latency

Formula preparations for treating insomnia				
Extract/Formula	Study design	Dosage	Mechanism	Result
Chaihu-Longgu-Muli decoction	*In vivo*, C57 BL/6J mice	2.36, 4.27, 9.45 g/kg/d, 4 weeks, i.g.	↑Orexin-A, ↑Orexin R1, ↑Orexin R2, ↑p-CaMKK2/AMPK, ↓NF-κB	↓Sleep disorder Improvement of circadian rhythm ↑Cognitive levels
Guben-Jiannao Ye	*In vivo*, APP/PS1 mice	8.97 g/kg, 3 months, i.p.	↑PI3K/Akt/mTOR, ↓amyloid-beta, ↑CLOCK, ↑BMAL1, ↑PER1	↑Sleep duration ↓Sleep latency
Huanglian Wendan Decoction	*In vivo*, SD rat	0.845, 1.69, 3.38 g/100 g/d, 30 days, i.p.	↑BDNF, ↑TrkB, ↑SCFA, ↑ IL-10, ↑Foxp3, ↓TNF-α, ↓IL-6	↑Sleep duration ↓Sleep latency
Shen Yuan	*In vivo*, ICR mice	1 g/kg/d, 14 days, i.g.	↑Trp, ↑5-HT, ↑Melatonin, ↑MT2, ↑Cry1	↑Sleep duration ↓Sleep latency
Shuangxia Decoction	*In vivo*, KM mice, SD rat, ICR mice	10 g/kg/d, 9 days, 7 g/kg/d, 7 days, 40 mg/kg/d, 7 days, i.p.	↑cAMP-PKA-CREB-Circadian, ↑GABA, ↓GLU, ↑5-HT, ↓CORT, ↓IL6, ↓TNF-α, ↓MDA, ↑CLOCK, ↑p-BMAL1/BMAL1, ↑PER3, ↑ZO-1, ↑Occludin, ↑Claudin	↑Sleep duration ↓Sleep latency
Suanzaoren Decoction	*In vivo*, ICR mice	3.6, 7.2, 14.4 g/kg/d, 24 days, i.p.	↓Orexin-A, ↓OX2R, ↓Glu/GABA, ↑5-HT, ↑5-HTR1A	↑Sleep duration ↓Sleep latency
Yiyin Anshen Granul	*In vivo*, ICR mice	4, 8, 16 g/kg/d, 31 days, i.g.	↑GABA, ↑5-HT, ↑DA, ↑GABAA, ↓Glu/GABA, ↓IL-6, ↑IL-1β, ↑Abundance and diversity of the intestinal microbiota	↑Sleep duration ↓Sleep latency Improvement of circadian rhythm
Ziziphi Spinosae Semen and Polygalae Radix	*In vivo*, KM mice,	2.5, 5, 10 g/kg/d, 7 days, i.g.	Restore gut microbiota, ↑5-HT, ↑Gas, ↑MT, ↑TNF-α, ↑IL-1β, ↓DA	↑Sleep duration ↓Sleep latency ↓Exercise time ↓Exercise distance

**TABLE 2:** Clinical research on natural products for treating insomnia.

Clinical research on natural products for treating insomnia			
Compound	Study design	Dosage	Result
*Aloysia citrodora* Paláu	Clinical trial, 71 individuals with insomnia	400 mg/d, 90 days	↑Sleep duration ↓Sleep latency
*Withania somnifera* (L.) Dunal	Clinical trial, 40 individuals with insomnia	600 mg/d, 8 weeks	↑Sleep duration ↓Sleep latency
*Morus *spp.	Clinical trial, 43 patients with sleep disorders	750 mg/d, 14 days	↑Sleep quality ↓Sleep latency ↑Mood after waking up
*Ocimum basilicum* L.	Clinical trial, 60 Menopausal women with sleep disorders	250 mg/d, 1 month	↑Sleep quality
*Poria cocos* (Schw.) Wolf	Clinical trial, 21 individuals with insomnia	800 mg/d	↑Sleep duration ↓Sleep awakening
*Rhodiola rosea* L./*Nelumbo nucifera* Gaertn.	Clinical trial, 20 individuals with insomnia	750 mg/d, 2 weeks	↑Sleep duration ↓Sleep latency
*Crocus sativus* L.	Clinical trial, 66 individuals with insomnia	15.5 mg/d, 6 weeks	↑Sleep duration ↓Sleep latency
Standardized lime peel supplement	Clinical trial, 80 individuals with insomnia	300 mg/d, 2 weeks	↑Sleep duration ↓Sleep latency
*Valeriana officinalis* L.	Clinical trial, 80 individuals with insomnia	200 mg/d, 8 weeks	↑Sleep duration ↓Sleep latency

### Mechanisms of natural products in treating insomnia

Natural products exert their therapeutic effects on insomnia by modulating neurotransmitter systems, particularly influencing γ-aminobutyric acid (GABA), serotonin (5-HT), and melatonin. Their low tolerance development and absence of withdrawal effects circumvent the dependency risks associated with benzodiazepines, while the biological compatibility of active metabolites significantly reduces the incidence of hepatic and renal function impairment. The following section surveys natural products with demonstrated anti-insomnia effects investigated within the past 5 years.

#### Jujuboside A

Jujuboside A is a triterpenoid saponin isolated from *Ziziphus jujuba* Mill. with anti-inflammatory, sedative, and hypnotic effects. Wang et al. identified doses of 9 and 18 mg/kg for Jujuboside A. Research has found that intervention with Jujuboside A at a dose of 18 mg/kg for 7 days significantly improved sleep latency and total sleep time in insomnia model mice. Its efficacy was comparable to that of the positive control drug Diazepam. Its mechanism of action involves upregulating GABA levels in the hippocampal region while inhibiting glutamate (Glu) and neuronal apoptosis, and simultaneously increasing the expression of GABA A and GABA B receptors, thereby protecting neurons and ameliorating insomnia (Wang et al., 2025). It has also been demonstrated that Jujuboside A ameliorates insomnia by restoring the mitochondrial membrane potential, inhibiting the opening of the mitochondrial permeability transition pore (mPTP), and reducing the accumulation of cytochrome c (Cyt c) in the neuronal cytoplasm (Zhang Z. et al., 2025). The present study not only confirmed the anti-insomnia efficacy of Jujuboside A, but moreover established the critical role of GABA in its therapeutic effect through employment of a GABA inhibitor.

#### *Humulus lupulus* L.

The perennial climbing plant hops (*Humulus lupulus* L.), yields a botanical drug commonly used to treat symptoms such as insomnia, anxiety, nervousness and fever. Lee et al. screened doses of hop extract at 50, 100, 150, and 200 mg/kg. Studies have demonstrated that 100 mg/kg hops extract significantly increases non-rapid eye movement (NREM) sleep and improves sleep quality in mice with insomnia for 1 h. The therapeutic mechanism involves activating GABA A receptors to enhance delta-wave sleep, upregulating 5-HT 1A receptor expression, and elevating GABA content in the brain, thereby contributing to its anti-insomnia effects. This study preliminarily validated the therapeutic efficacy of hop extract for insomnia and clarified the crucial role of GABA in the treatment process through GABA antagonists. However, it lacked a positive drug control (Lee et al., 2025).

#### *Nelumbo nucifera* Gaertn

*Nelumbo nucifera* Gaertn., an aquatic plant of the Nelumbonaceae family, possesses medicinal values including hemostatic and anticancer effects. Lotus rhizome (LE) constitutes a significant component, and its efficacy in treating insomnia remains unexplored. Ahn et al. screened LE at dosage ranges of 80, 100, 120, and 150 mg/kg. Studies revealed that administration of 150 mg/kg LE to mice for 1 h significantly prolonged non-rapid eye movement sleep in insomnia-model animals. This effect was comparable to that of the positive drug alprazolam. The mechanism involves activating GABA A receptors and increasing GABA concentration in the brain, thereby alleviating insomnia. This study featured a comprehensive design, not only validating LE’s therapeutic efficacy for insomnia but also confirming GABA’s crucial role in LE’s mechanism of action through GABA antagonists (Ahn et al., 2022).

#### Lavender

Lavender (*Lavandula angustifolia* Mill.), a semi-shrub or dwarf shrub belonging to the Lamiaceae family, exhibits antibacterial and anti-inflammatory pharmacological properties. Ren et al. designed a precise odour delivery system to administer varying concentrations of lavender essential oil (LEO) to the nasal tips of mice for therapeutic purposes. In a study using mice with PCPA-induced insomnia models, intranasal administration of 25.0% LEO for 2 h significantly shortened the latency to non-rapid eye movement (NREM) sleep and increased the total amount of NREM sleep. The molecular mechanism involves sleep improvement through the olfactory pathway and targeting central amygdala GABA neurons. This study preliminarily validated LEO’s anti-insomnia effects, though it lacked a positive drug control and rescue experiments to definitively establish GABA’s pivotal role (Ren et al., 2025).

#### Mulberry leaf

Mulberry leaf (*Morus alba* L.), the dried leaf of the dicotyledonous mulberry tree, is a homologous food and medicine commonly used clinically to treat insomnia. Purified flavone from mulberry leaves (MLF) is its key active metabolite. Li et al. screened MLF at doses of 50, 100, and 200 mg/kg. Studies have found that daily intervention with 200 mg/kg of MLF for 7 days significantly reduced sleep latency in insomniac mice, prolonged their sleep duration, and increased their sleep efficiency. Its effects were comparable to those of the positive control drug Estazolam. The mechanism involves increasing the release of GABA and serotonin (5-HT) in the serum, hypothalamus, and hippocampus. This study preliminarily validated the therapeutic efficacy of MLF for insomnia; however, it lacked rescue experiments to further clarify the role of GABA in the treatment (Li et al., 2025).

#### *Panax ginseng* C. A. Mey

*Panax ginseng* C. A. Mey. is a perennial plant of the genus Panax, with health-promoting effects and used to treat insomnia and cardiovascular diseases. Lin et al. screened P. ginseng alcohol extract (GAE) at doses of 7.5, 15, and 30 mg/kg. It is widely utilized globally. Studies have found that daily administration of GAE (30 mg/kg) to rats for 28 days significantly prolonged sleep duration, improved cognitive function, prevented hippocampal neuronal damage, increased the number of Nissl bodies, ameliorated aging and sleep markers, and enhanced LC3A/B expression in autophagosomes and neurons. Its effects were comparable to those of the positive control drug eszopiclone. The mechanism of action involves reducing orexin secretion in the hypothalamus and interacting with orexin receptors, thereby inhibiting the PI3K/Akt/mTOR signaling network and activating autophagy to protect neurons, thus alleviating insomnia. This study preliminarily validated GAE’s efficacy for insomnia and its potential mechanism of action, though rescue experiments were lacking to definitively establish orexin’s role in treatment (Lin et al., 2025).

#### Periostracum cicadae

Periostracum cicadae, the exuviae of Cicadidae, is an animal-derived medicinal material commonly used clinically to treat nocturnal crying in children and insomnia. The doses of cicada moult extract screened by Wang et al. comprised 18.272 and 4.568 mg/mL. Studies have found that daily intervention with Periostracum cicadae extract (18.272 mg/mL) for 7 days significantly reduced the total distance traveled and mean speed, while increasing immobility time in insomnia-model rats during the open field test. This effect was comparable to that of the positive drug diazepam. The mechanism of action involves upregulating the expression of 5-HT1A, BDNF, and DARPP-32 proteins, thereby exerting its therapeutic effect against insomnia. The experimental design of this study is relatively comprehensive, though it lacks a rescue experiment to definitively establish the role of 5-HT in the therapeutic process (Wang et al., 2024).

#### Rosemary

Rosemary (*Rosmarinus officinalis* L.), a plant native to the Mediterranean Basin, is widely used in traditional medicine to treat insomnia, depression, and anxiety. Li et al. screened rosemary extracts at doses of 10, 12.5 and 15 g/kg. Studies have found that 15 g/kg rosemary extract administered for six consecutive days significantly improved circadian rhythms in insomnia-model rats, matching the efficacy of the positive control drug diazepam. Furthermore, plasma levels of 5-HT, GABA, and DA were markedly elevated in these rats, while hippocampal protein levels of 5-HT-1A R, PKA, GABA A, cAMP, and ADCY5 showed varying degrees of increase. Its mechanism of action may involve GABA and 5-HT pathways. This study provides preliminary evidence that rosemary hydrosol may alleviate insomnia, though further rescue experiments are required to substantiate the role of GABA in this process (Li T. et al., 2024).

#### Sedum kamtschaticum

*Sedum kamtschaticum* Fisch., a perennial plant, possesses anti-inflammatory, antioxidant, and anxiolytic effects. Kim et al. screened an ethanol extract (ESK) of *Sedum kamtschaticum* at doses of 3, 10, and 30 mg/kg. Studies have found that administering 10 or 30 mg/kg ESK to insomniac mice for 1 h significantly reduced their sleep latency and prolonged total sleep time, with effects comparable to those of the positive control drug diazepam. The mechanism of action involves activating adenosine A_2A_ receptors and promoting the release of GABA in the hypothalamus, thereby treating insomnia. This study has preliminarily established the therapeutic efficacy of ESK extract; however, Western blot results are lacking to validate the experimental findings (Kim Y. S. et al., 2024).

### Mechanisms of traditional Chinese medicine formulations in treating insomnia

Traditional Chinese Medicine formulations boast a long history of application, having developed unique path mechanism adaptation principles through nearly two millennia of clinical optimization. Their use in insomnia treatment has likewise demonstrated notable efficacy. The core advantage of TCM formulations lies in their adherence to the “sovereign-minister-assistant-courier” compatibility principle with natural products, achieving multi-target synergistic integration to reconstruct regulatory networks encompassing neurotransmitters (GABA/5-HT/orexin) and immune factors (TNF-α/IL-1β). Below are studies on insomnia-treating formulations conducted in the past 5 years.

#### Chaihu-Longgu-Muli decoction

Chaihu-Longgu-Muli Decoction is a classic clinical formula widely used to treat neuropsychiatric disorders such as insomnia, anxiety, and dementia. The CLMD formula contained the following: *Bupleurum chinense* DC. (root) (12 g), *Fossilia Ossia Mastodi* (skeleton) (4.5 g), *Scutellaria baicalensis* Georgi (root) (4.5 g), *Zingiber officinale Roscoe* (root) (4.5 g), *Panax ginseng C.* A. Mey. (root) (4.5 g), *Cinnamomum cassia* (L.) J.Presl (bark) (4.5 g), *Poriacocos (Schw.)* Wolf (nucleus) (4.5 g), *Pinellia ternata* (Thunb.) Makino (tuber) (6g), *Rheum palmatum* L. (root) (6 g), *Ostrea gigas Thunberg* (shell) (4.5 g), and *Ziziphus jujuba* Mill. (fruit) (4.5 g). Studies have demonstrated that administering 9.45 g/kg/d Chaihu-Longgu-Muli Decoction to mice for 4 weeks significantly improved circadian rhythms and sleep-wake patterns. The therapeutic mechanism involves upregulating orexin-A expression and CaMKK2/AMPK phosphorylation levels, thereby further inhibiting the downstream NF-κB signaling pathway. This cascade ultimately alleviates inflammatory dysregulation in both central and peripheral systems, contributing to its anti-insomnia effects. This study preliminarily validated the therapeutic efficacy and potential mechanism of action of Chaihu-Longgu-Muli decoction in treating insomnia. However, it lacked a positive control drug and did not conduct rescue experiments to clarify the role of orexin-A in the treatment (Cao et al., 2023).

#### Guben-Jiannao Ye

Guben-Jiannao Ye has a long history of use in traditional Chinese medicine for treating learning and memory impairments and senile insomnia. The Guben-Jiannao Ye formula contained the following: *Codonopsis pilosula* (Franch.) Nannf. (root) (15 g), *Wolfiporia cocos* (Schw.) Ryv. &Gibn. (nucleus) (12 g), *Lycium barbarum* L. (fruit) (15 g), *Crataegus pinnatifida* Bunge. (fruit) (12 g), *Ziziphus jujuba* Mill. (fruit) (15 g). A study demonstrated that intervention with 8.97 g/kg of Guben-Jiannao Ye for 3 months in insomnia model mice significantly enhanced cognitive ability, altered clock gene expression patterns, improved non-rapid eye movement (NREM) and rapid eye movement (REM) sleep, and alleviated insomnia. Its effects are similar to those of the positive control melatonin. The mechanism involves activating the PI3K/Akt/mTOR signaling pathway and reducing amyloid-beta deposition in the hippocampus, thereby exerting its effects. Although this study provided preliminary validation of the therapeutic efficacy of Guben-Jiannao Ye for insomnia, no rescue experiments were conducted to definitively establish the role of the PI3K/Akt/mTOR pathway in the treatment (Mao et al., 2024).

#### Huanglian Wendan decoction

Huanglian Wendan Decoction is a traditional Chinese medicine with effects including regulating qi, clearing dampness and resolving phlegm, calming the spirit, and arresting restlessness. The Huanglian Wendan Decoctio contained the following: *Glycyrrhiza uralensis* Fisch (root) (5 g), *Coptis chinensis* Franch (root) (6 g), *Paeonia lactiflora* Pall (root) (15 g), *Atractylodes macrocephala* Koidz (root) (15 g), *Angelica sinensis* (Oliv.) Diels (root) (15 g), *Pinellia ternate* (Thunb.) Breit (tuber) (15 g), *Alisma orientale* (Sam.) Juzep (tuber) (15 g), *Citrus reticulata* Blanco (fruit) (15 g), *Citrus aurantium* L (fruit) (15 g), *Bupleurum chinense* DC (root) (15 g), *Bambusa tuldoides* Munro (interlayer) (15 g), *Poria cocos* (Schw.) Wolf (nucleus) (15 g). Research has found that intervention with Huanglian Wendan Decoction at a dose of 3.38 g/100 g for 1 month significantly enhanced learning and memory abilities, lowered sleep latency, and prolonged total sleep time in insomnia model rats. Its mechanism of action involves restoring the richness and diversity of the gut microbiota in insomnia model rats by increasing the expression levels of BDNF and TrkB in the hippocampus, and promoting the production of SCFAs, thereby reducing neuroinflammation and alleviating insomnia. The experimental design of this study is comprehensive. Although lacking a positive drug control, it employed a TrkB receptor agonist to validate the target action. However, as Huanglian Wendan Decoction primarily functions by elevating p-TrkB levels, the study should have utilised TrkB receptor inhibitors for rescue experiments (Shi et al., 2025).

#### Shen Yuan

*Panax ginseng* C. A. Mey. (root) and *Polygala tenuifolia* Willd. (root) are perennial plant used to treat neurasthenia, cough, and digestive disorders. Clinically, Ginseng Radix and Polygalae Radix (Shen Yuan) are commonly used to treat insomnia, depression, and other psychiatric disorders. Research has demonstrated that purifying Ginseng Radix and Polygalae Radix in a 3:2 ratio yields a 1 g/kg SY extract, which was administered to mice for 14 days. Findings revealed that the SY extract significantly reduced sleep latency and increased sleep duration in the mice. Its effects are similar to those of the positive drug diazepam. The mechanism of action involves activating the Trp/5-HT/melatonin pathway to exert hypnotic effects, enhancing the synthesis of 5-hydroxytryptamine (5-HT) and melatonin, and consequently increasing the expression of melatonin receptor subtype 2 (MT2) and cryptochrome 1 (Cry1). Although this study preliminarily validated the therapeutic efficacy of SY extract for insomnia, no rescue experiments were conducted to clarify the role of 5-HT in the treatment (Xia et al., 2024).

#### Shuangxia decoction

Shuangxia Decoction is composed of *Pinellia ternata* (Thunb.) Makino (tuber) and *Prunella vulgaris* L. (fruit) It is a traditional Chinese botanical drug formula used to treat insomnia. Studies have found that daily intervention with 10 g/kg of Shuangxia Decoction for 9 days alleviates insomnia by increasing cortical serotonin (5-HT) levels while decreasing dopamine and norepinephrine levels (Sun et al., 2020). Another study demonstrated that intervention with Shuangxia Decoction at 7 g/kg/day for 7 days significantly shortened sleep latency and prolonged total sleep time in rats. Its therapeutic effect is comparable to that of the positive control drug eszopiclone. Concurrently, Shuangxia Decoction significantly reduced serum levels of CORT, IL-6, TNF-α, and MDA, decreased hypothalamic glutamate (Glu) levels, and increased levels of GABA and serotonin (5-HT). The mechanism of action may involve activation of the cAMP-PKA-CREB-circadian rhythm pathway, improve the rhythmicity of the biological clock, and thereby promote sleep (Zhang et al., 2024). Furthermore, studies have found that daily intervention with 40 mg/kg of rosmarinic acid (a representative formula of Shuangxia Decoction) for 7 days in mice alleviates insomnia by upregulating tight junction proteins ZO-1, Occludin, and Claudin; modulating the Nrf2 signaling pathway; eliminating intestinal ROS accumulation; and ameliorating oxidative stress-induced gut microbiota dysbiosis (Liu et al., 2025). The aforementioned studies have demonstrated the anti-insomnia efficacy of Shuangxia Decoctio from multiple perspectives; however, rescue experiments are lacking to explicitly confirm the pivotal role of inflammation and oxidative stress in the mechanism of action.

#### Suanzaoren decoction

Suanzaoren Decoction, a traditional Chinese botanical formula used to treat insomnia, has a history spanning thousands of years. Its constituent components are *Ziziphus jujuba* Mill. (fruit) (Suanzaoren), *Dendrobium officinale* Kimura & Migo (rhizome) (Tiepishihu), *Conioselinum anthriscoides “Chuanxiong”* (rhizome) (Chuanxiong), *Poria cocos* (Schw.) Wolf (nucleus) *and Glycyrrhiza uralensis* Fisch. ex DC. (root) (Gancao), blended in a 5:2:2:2:1 ratio. Studies have found that administering Suanzaoren Decoction at a dose of 14.4 g/kg/day to mice with PCPA-induced insomnia shortened the sleep latency, prolonged the sleep duration, and improved circadian rhythm disruptions in the insomniac mice. Its effects are similar to those of the positive drug diazepam. The mechanism of action involves downregulating serum Orexin-A levels and hypothalamic OX2R expression, decreasing the Glu/GABA ratio, and increasing the levels of 5-HT and 5-HTR1A proteins in the hypothalamus, thereby exerting its therapeutic effects against insomnia (Dong et al., 2021). Another study also confirmed that 14 g/kg/day of Suanzaoren Decoction can improve sleep in mice and increase the levels of 5-HT, GABA, and NE in the hippocampus. This study provided preliminary validation of the therapeutic efficacy of Suanzaoren Decoction in treating insomnia; however, no rescue experiments were conducted to elucidate the role of Orexin-A/GABA in the treatment mechanism (Yan et al., 2023).

#### Yiyin Anshen Granule

Yiyin Anshen Granule, a traditional Chinese medicinal formula, is clinically used to treat insomnia, fatigue, excessive dreaming, nocturnal awakenings, and menopausal syndrome. Its constituent components are *Ziziphus jujuba* Mill. (fruit) (7.5 g), *Polygala tenuifolia* Willd. (root) (7.5 g), *Schisandra chinensis* (Turcz.) Baill. (fruit) (3 g), *Rehmannia glutinosa* Libosch. (tuber) (15 g), *Dioscorea opposita* Thunb. (rhizome) (15 g), *Ophiopogon japonicus* D.Don (tuber) (12 g), *Poria cocos* (Schw.) Wolf (nucleus) (12 g). Studies have demonstrated that administration of 16 g/kg Yiyin Anshen Granule for 31 days significantly shortened sleep latency and prolonged sleep duration in insomnia-model mice, effectively ameliorating circadian rhythm disruption. Its effects are similar to those of the positive drug diazepam. The mechanism involves increasing 5-HT, GABA, and GABA A Rα1 expression while reducing the Glu/GABA ratio, thereby improving levels of inflammatory factors IL-6 and interleukin-1β, and enhancing the richness and diversity of intestinal microbiota to alleviate insomnia. This study possesses extensive data on gut microbiota and GABA-related parameters, yet lacks rescue experiments under antibiotic administration. Consequently, the precise role of gut microbiota in the therapeutic effects of Yiyin Anshen Granule remains to be further elucidated (Zhang C. et al., 2025).

#### Ziziphi Spinosae Semen and Polygalae Radix

Ziziphi Spinosae Semen (ZSS) is the dried ripe seed of *Ziziphus jujuba* Mill. Polygalae Radix (PR) is the dried root of *Polygala tenuifolia* Willd. Ziziphi Spinosae Semen and Polygalae Radix (ZSS-PR) is a traditional Chinese herb pair commonly used clinically to treat anxiety and insomnia. Research has found that purifying ZSS and PR in a 2:1 ratio yields 3.45 mg/kg of ZSS-PR. ZSS-PR significantly reduced sleep latency and prolonged sleep duration in insomnia-model mice (Luo et al., 2020). Daily intervention with ZSS-PR (10.0 g/kg) for 7 days significantly increased locomotor time and distance, and ameliorated pathological damage in the cerebral cortex and hippocampus of insomnia-model mice. Its effects are similar to those of the positive drug diazepam. The underlying mechanism involves restoring the relative abundance of the gut microbiota and altering phenylalanine, tyrosine and tryptophan biosynthesis, as well as glutamine and glutamate metabolism, thereby elevating levels of 5-hydroxytryptamine (5-HT), gastrin (Gas), melatonin (MT), TNF-α, and interleukin-1 beta (IL-1β), while reducing dopamine (DA) levels. This study demonstrated the therapeutic efficacy of ZSS-PR for insomnia, but did not employ antibiotics to further elucidate the role of the gut microbiota in this therapeutic effect (Ren et al., 2024).

### Clinical research on natural products for treating insomnia

In clinical investigations of natural products, various natural extracts have demonstrated remarkable efficacy. These findings underscore the potential of natural products in modulating insomnia, particularly with a lower incidence of adverse events, offering gentler therapeutic alternatives for patients. Below are clinical studies on natural products for insomnia treatment conducted over the past 5 years.

#### *Aloysia citrodora* Paláu

*Aloysia citrodora* Paláu, commonly known as “lemon verbena,” possesses multiple biological activities, including antioxidant, anti-anxiety, neuroprotective, anticancer, antibacterial, and sedative effects. A randomized double-blind controlled trial involving 71 insomnia patients found that daily intake of 400 mg of lemon verbena for 90 days significantly improved sleep latency and sleep efficiency, reduced wake after sleep onset, and significantly increased nocturnal plasma melatonin levels (Pérez-Piñero et al., 2024). The study employed an adequate sample size and demonstrated good safety throughout the experimental process, thereby conclusively establishing the efficacy of lemon verbena in treating insomnia.

#### Ashwagandha

Ashwagandha *(Withania somnifera* (L.) Dunal.) possesses a broad spectrum of pharmacological effects, including anti-anxiety, hypotensive, sedative, anti-inflammatory, antitumor, antifungal, hematopoietic, and cardiopulmonary-enhancing activities. A clinical study involving 40 patients with insomnia and non-insomnia controls demonstrated that 600 mg/day of Ashwagandha root extract significantly improved HAM-A (Hamilton Anxiety Rating Scale) scores, mental alertness, and sleep quality. Furthermore, significant improvements were observed in sleep onset latency, total sleep time, and sleep efficiency (Langade et al., 2021). This study provides preliminary evidence for the therapeutic efficacy of Ashwagandha root extract in treating insomnia, though it lacks assessment of hormonal or neurotransmitter levels within patients.

#### Mulberry leaf

Mulberry leaf is the leaf of *Morus* spp. and possesses functions in alleviating hypertension and treating cough. A clinical trial involving 43 cases with sleep difficulties demonstrated that daily intake of 750 mg Mulberry leaf extract plus 120 mg tryptophan for 14 days significantly reduced sleep onset latency, improved sleep quality, and enhanced morning mood in adults. Additionally, the extract significantly reduced the postprandial glucose response at 1 h after dinner (Soon et al., 2025). This study provides preliminary evidence for the therapeutic efficacy of mulberry leaf in treating insomnia, though it lacks assessment of neurotransmitter levels within patients.

#### *Ocimum basilicum* leaf

*Ocimum basilicum* L., an annual herb of the Lamiaceae family, possesses therapeutic effects including memory enhancement and treatment for headaches, bloating, and related conditions. A clinical trial involving 60 menopausal women demonstrated that daily intake of 250 mg *Ocimum basilicum* leaf extract for 1 month significantly improved sleep quality and reduced insomnia symptoms (Karimi et al., 2023). This study provides preliminary validation of the therapeutic efficacy of *Ocimum basilicum* leaf extract for insomnia; however, it lacks assessment of sleep latency, sleep efficiency, and wakefulness time following sleep onset.

#### Poria cocos

*Poria cocos* is the dried sclerotium of the fungus *Poria cocos* (Schw.) Wolf (Polyporaceae family), possessing therapeutic effects including anti-tumor, anti-inflammatory, antioxidant, and memory-enhancing properties. A clinical trial involving 21 insomnia patients demonstrated that daily intake of 800 mg *Poria cocos* extract significantly increased total sleep duration and reduced wakefulness levels during sleep (Kim et al., 2023). This study provides preliminary evidence for the efficacy of Poria cocos extract in treating insomnia, though the sample size was somewhat inadequate.

#### *Rhodiola rosea* L.

*Rhodiola rosea* L. is a perennial herb commonly known as rose root. *Nelumbo nucifera* Gaertn. is an aquatic herb of the lotus genus, also known as lotus. Both plants possess anti-anxiety and sedative effects. Research has combined extracts from these two plants to obtain a novel TCM formula mixture designated RNE. A clinical study involving 20 insomnia patients found that intervention with RNE at a dose of 750 mg/day for 2 weeks significantly improved sleep quality, reduced wake after sleep onset, and increased sleep efficiency in the patients, with demonstrating good safety (Kim Y. et al., 2024). This study provides preliminary evidence for the efficacy of RNE extract in treating insomnia, though the sample size was somewhat inadequate.

#### Saffron

Saffron, the dried stigmas of *Crocus sativus* L., exhibits diverse pharmacological effects, including anti-fatigue, anti-aging, and anticancer activities. A randomized, double-blind, controlled clinical trial involving 66 patients with insomnia demonstrated that 15.5 mg/day of saffron extract, following 6 weeks of intervention, significantly improved time in bed and difficulty falling asleep in the insomnia patients. Assessment using the PSQI questionnaire revealed that the saffron extract significantly improved sleep quality, sleep latency, and sleep duration, while the placebo failed to alter these parameters (Pachikian et al., 2021). This study provides preliminary evidence of the efficacy of saffron extract in treating insomnia. Further investigation into neurotransmitter levels within patients would yield greater value.

#### Standardized lime peel supplement

Standardized lime peel supplement (SLPS) possesses potential sedative-hypnotic effects. A clinical trial involving 80 subjects demonstrated that daily intake of 300 mg SLPS for 2 weeks significantly reduced sleep latency, wakefulness after sleep onset, and total wake time, while improving sleep efficiency and total sleep time. No severe adverse reactions occurred in the SLPS group during the intervention period (Kim et al., 2025). This study validated the therapeutic efficacy of SLPS for insomnia, though it lacked assessment of neurotransmitter levels within patients.

#### Valerian

Valerian extract (*Valeriana officinalis* L.) possesses antioxidant, antibacterial, anti-inflammatory, sedative, cytoprotective, and neuroprotective activities. A randomized, double-blind, controlled clinical trial involving 80 patients with insomnia demonstrated that intervention with 200 mg/day of valerian extract for 8 weeks significantly improved sleep latency, actual sleep time, and sleep efficiency in the insomnia patients (Chandra Shekhar et al., 2024). Furthermore, valerian extract did not cause any significant toxicological changes in skeletal and visceral examinations *in vivo* (Bao et al., 2024). This study validated the therapeutic efficacy of valerian extract for insomnia, though it did not conduct further testing on neurotransmitter levels within patients.

**FIGURE 1:**
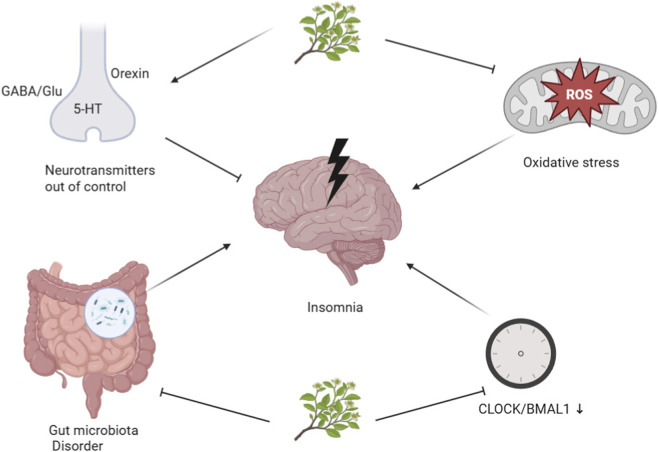
Mechanism diagram of natural products intervening in insomnia. Schematic diagram created with BioRender.com.

In summary, natural products demonstrate favorable therapeutic effects for insomnia. Their mechanisms include: rebalancing excitatory-inhibitory neurotransmission through concurrent GABA potentiation and glutamate (GLU) suppression; serotonergic optimization *via* synergistic 5-HT modulation; hypothalamic orexinergic stabilization; remodeling the gut-brain axis by regulating intestinal microbiota to increase GABA production while reducing glutamate-secreting *Bacteroides*; and circadian entrainment *via* CLOCK/BMAL1 amplitude enhancement. Moreover, natural products achieve validated clinical outcomes in insomnia treatment with a good safety profile. Please refer to Figure 1 for details.

## Comparison between natural products and traditional medicines

Natural products exhibit comparable sleep-enhancing efficacy to synthetic counterparts, albeit with distinct pharmacodynamic profiles characterized by delayed onset. Paradoxically, conventional agents like benzodiazepines—while providing rapid symptomatic relief—entail significant iatrogenic risks including receptor desensitization and physiological dependence upon chronic administration (Darke, 1994; Stewart and Westra, 2002). However, natural products including *Aloysia citrodora* Paláu and Ashwagandha have shown favorable outcomes in clinical trials, enhancing both sleep quality and sleep onset latency in patients (See Tables 1 and 2 for details) (Pérez-Piñero et al., 2024; Langade et al., 2021). However, some clinical studies still suffer from small sample sizes, short research periods, and a lack of long-term safety data, which remain areas requiring improvement. Benzodiazepines are frequently associated with adverse effects such as somnolence, memory impairment, and dependence, which significantly compromise patients’ quality of life and may contribute to substance misuse (Cooke et al., 2020). Although natural products exhibit relatively slower therapeutic onset, they offer favourable safety profiles and contribute to improved overall mental health, particularly in alleviating anxiety and depressive symptoms, rendering them potentially advantageous for long-term sleep management (Powers-James et al., 2024). For instance, research demonstrates that sleep supplements containing natural metabolites effectively improve sleep quality without significant safety concerns (Kim et al., 2025). Furthermore, growing evidence from intensified safety investigations suggests that appropriately dosed botanicals can safely and effectively enhance sleep quality without inducing dependence (Moulin et al., 2023). Consequently, whereas conventional hypnotics offer expedient symptomatic relief for acute sleep disruption, natural products demonstrate superior therapeutic sustainability in chronic insomnia management through pharmacologically adaptive modulation of sleep-wake circuitry and reduced adverse event liability.

## Future research directions and challenges

In research on natural products for insomnia treatment, identifying active metabolites and elucidating their structure-activity relationships present critical challenges. Although existing studies demonstrate the sleep-improving effects of various plant extracts, the specific natural products responsible for soporific actions and their mechanisms of action require further investigation (Rao et al., 2022). For instance, research indicates that flavonoids in certain plants may influence sleep by modulating neurotransmitter levels (Li et al., 2025), yet plant extracts from different sources contain varying active metabolites (Tokin et al., 2021). Consequently, systematic compositional analysis using modern molecular biology and chemical profiling techniques is essential to identify principal bioactive natural products and explore their structure-activity relationships (Aghelan et al., 2023; Zhao et al., 2024; Zhao M. et al., 2023; Zhao et al., 2025). Furthermore, with advancements in high-throughput screening technologies, computational biology approaches can be employed to predict bioactive metabolites in plants, thereby accelerating development of novel hypnotic agents (Jiang et al., 2024). These laboratory techniques enable the rational design of natural compound combinations, a strategy that corrects neurochemical imbalances at their source. This approach enhances sleep maintenance efficiency in refractory patients without residual sedative effects. However, while promoting development in this field, we must also be wary of potential differences in perspectives and findings between different studies. The efficacy and safety of natural products are influenced by a variety of factors, including extraction methods, dosage, and duration of use. Therefore, when integrating results from different studies, it is important to consider the heterogeneity of study designs, reasonably assess the credibility of each study, and form a more comprehensive understanding based on this assessment.

Based on the primary therapeutic pathways of natural products, we propose a four-step precision translation framework: (1) Establishing compound-specific target engagement thresholds through neuroimaging-based target validation in patient-derived organoids; (2) Enhancing natural product brain bioavailability *via* nano-CNS delivery using transferrin receptor-functionalised blood-brain barrier-penetrating exosomes; (3) triggering real-time transdermal administration *via* smart microneedle patches to maintain patient neurotransmitter equilibrium; (4) employing a hybrid trial design for practical clinical validation in refractory insomnia cohorts, initially determining patient-specific dosing parameters followed by randomised washout periods measuring rebound insomnia incidence as the primary endpoint. This vertically integrated approach translates mechanistic research into clinically actionable solutions, offering greater convenience and efficacy compared to conventional therapies.

Despite growing interest in natural products for insomnia treatment, standardization of clinical research remains a significant challenge. Existing studies predominantly employ disparate evaluation criteria and research designs, compromising the reliability and comparability of findings. To enhance research quality, future clinical trials should adopt unified assessment standards, including validated tools such as the Insomnia Severity Index (ISI) for symptom evaluation and quality-of-life scales (Morin et al., 2024). Concurrently, implementing stringent inclusion/exclusion criteria ensures cohort uniformity critical for enhancing clinical trial validity; synergistically, leveraging artificial intelligence-enabled analytics facilitates the integration of multi-source data with unprecedented mechanistic depth, generating clinically translatable evidence for therapeutic applications.

## Conclusion

Collectively, the considerable potential demonstrated by natural products in insomnia management foreshadows future diversified and personalized therapeutic strategies. Current therapeutic approaches of natural products for insomnia management primarily operate through four mechanisms: (1) restoring excitatory-inhibitory neurotransmission balance *via* GABA modulation; (2) synergistically ameliorating insomnia through combined regulation of inflammation and oxidative stress; (3) remodeling the brain-gut axis by modulating gut microbiota to alleviate insomnia; (4) improving sleep disorders through circadian rhythm regulation by targeting CLOCK/BMAL1 proteins. Nevertheless, further research remains imperative to elucidate active metabolites and mechanisms of action of natural products, thereby providing more robust scientific foundations for clinical implementation. Moreover, ongoing research and clinical collaboration are of paramount importance. Through integrative utilization of both natural products and conventional medications, enhanced therapeutic regimens with improved safety and efficacy profiles may be developed for insomnia patients, ultimately ameliorating their quality of life. Consequently, promoting collaborative research in this domain will yield significant implications for the future advancement of insomnia treatment.
